# Differential effectiveness of *Serratia plymuthica *IC1270-induced systemic resistance against hemibiotrophic and necrotrophic leaf pathogens in rice

**DOI:** 10.1186/1471-2229-9-9

**Published:** 2009-01-22

**Authors:** David De Vleesschauwer, Leonid Chernin, Monica M Höfte

**Affiliations:** 1 Laboratory of Phytopathology, Faculty of Bioscience Engineering, Ghent University, Coupure links 653, B-9000 Gent, Belgium; 2 Department of Plant Pathology and Microbiology, Faculty of Agricultural, Food and Environmental Quality Sciences, the Hebrew University of Jerusalem, P.O.B. 12, Rehovot 76100, Israel

## Abstract

**Background:**

Induced resistance is a state of enhanced defensive capacity developed by a plant reacting to specific biotic or chemical stimuli. Over the years, several forms of induced resistance have been characterized, including systemic acquired resistance, which is induced upon localized infection by an avirulent necrotizing pathogen, and induced systemic resistance (ISR), which is elicited by selected strains of nonpathogenic rhizobacteria. However, contrary to the relative wealth of information on inducible defense responses in dicotyledoneous plants, our understanding of the molecular mechanisms underlying induced resistance phenomena in cereal crops is still in its infancy. Using a combined cytomolecular and pharmacological approach, we analyzed the host defense mechanisms associated with the establishment of ISR in rice by the rhizobacterium *Serratia plymuthica *IC1270.

**Results:**

In a standardized soil-based assay, root treatment with IC1270 rendered foliar tissues more resistant to the hemibiotrophic pathogen *Magnaporthe oryzae*, causal agent of the devastating rice blast disease. Analysis of the cytological and biochemical alterations associated with restriction of fungal growth in IC1270-induced plants revealed that IC1270 primes rice for enhanced attacker-induced accumulation of reactive oxygen species (ROS) and autofluorescent phenolic compounds in and near epidermal cells displaying dense cytoplasmic granulation. Similar, yet more abundant, phenotypes of hypersensitively dying cells in the vicinity of fungal hyphae were evident in a gene-for-gene interaction with an avirulent *M. oryzae *strain, suggesting that IC1270-inducible ISR and R protein conditioned effector-triggered immunity (ETI) target similar defense mechanisms. Yet, this IC1270-inducible ISR response seems to act as a double-edged sword within the rice defense network as induced plants displayed an increased vulnerability to the necrotrophic pathogens *Rhizoctonia solani *and *Cochliobolus miyabeanus*. Artificial enhancement of ROS levels in inoculated leaves faithfully mimicked the opposite effects of IC1270 bacteria on aforementioned pathogens, suggesting a central role for oxidative events in the IC1270-induced resistance mechanism.

**Conclusion:**

Besides identifying ROS as modulators of antagonistic defense mechanisms in rice, this work reveals the mechanistic similarities between *S. plymuthica*-mediated ISR and R protein-dictated ETI and underscores the importance of using appropriate innate defense mechanisms when breeding for broad-spectrum rice disease resistance.

## Background

Plants have evolved a powerful immune system to resist their potential colonization by microbial pathogens and parasites. Over the past decade, it has become increasingly clear that this innate immunity is, in essence, composed of two interconnected branches, termed PAMP-triggered immunity (PTI) and effector-triggered immunity (ETI) [1,2]. PTI is triggered by recognition of pathogen- or microbial-associated molecular patterns (PAMPs/MAMPs), which are conserved molecular signatures decorating many classes of microbes, including non-pathogens. Perception of MAMPs by pattern recognition receptors (PRRs) at the cell surface activates a battery of host defense responses leading to a basal level of resistance [3]. As a result of the evolutionary arms-race between plants and their intruders, many microbial pathogens acquired the ability to dodge PTI-based host surveillance via secretion of effector molecules that intercept MAMP-triggered defense signals [4]. In turn, plants have adapted to produce cognate R-(resistance) proteins by which they recognize, either directly or indirectly, these pathogen-specific effector proteins, resulting in a superimposed layer of defense variably termed effector-triggered immunity (ETI), gene-for-gene resistance or *R*-gene-dependent resistance [1].

In many cases, effector recognition culminates in the programmed suicide of a limited number of challenged host cells, clearly delimited from the surrounding healthy tissue. This hypersensitive response (HR) is thought to benefit the plant by restricting pathogen access to water and nutrients and is correlated with an integrated set of physiological and metabolic alterations that are instrumental in impeding further pathogen ingress, among which a burst of oxidative metabolism leading to the massive generation of reactive oxygen species (ROS) [5,6]. Apart from local immune responses, ETI-associated HR formation also mounts a long-distance immune response termed systemic acquired resistance (SAR), in which naïve tissues become resistant to a broad spectrum of otherwise virulent pathogens [7]. It should be noted, however, that PTI, when activated by PAMPs that activate the SA signaling pathway, can trigger SAR as well [8].

An archetypal inducible plant defense response, SAR requires endogenous accumulation of the signal molecule salicylic acid (SA) and is marked by the transcriptional reprogramming of a battery of SA-inducible genes encoding pathogenesis-related (PR) proteins. By contrast, there is ample evidence for induced disease resistance conditioned by molecules other than SA, as illustrated by rhizobacteria-mediated induced systemic resistance [ISR; [9]]. ISR, which delivers systemic protection without the customary pathogenesis-related protein induction, is a resistance activated upon root colonization by specific strains of plant growth-promoting rhizobacteria (PGPRs) [10]. In a series of seminal studies using the reference strain *Pseudomonas fluorescens *WCS417r, Pieterse and associates [11-13] demonstrated that, at least in Arabidopsis, ISR functions independently of SA, but requires components of the jasmonic acid (JA) and ethylene (ET) response pathways. Even though colonization of the roots by ISR-triggering bacteria leads to a heightened level of resistance against a diverse set of intruders, often no defense mechanisms are activated in aboveground plant tissues upon perception of the resistance-inducing signal. Rather, these tissues are sensitized to express basal defense responses faster and/or more strongly in response to pathogen attack, a phenomenon known as priming [14]. As demonstrated recently, priming of the plant's innate immune system confers broad-spectrum resistance with minimal impact on seed set and plant growth [15]. Hence, priming offers a cost-efficient resistance strategy, enabling the plant to react more effectively to any invader encountered by boosting infection-induced cellular defense responses [16,17].

In contrast to the overwhelming amount of information on inducible defenses in dicotyledonous plant species, our understanding of the molecular mechanisms underpinning induced disease resistance in rice (*Oryza sativa*) and other cereals is still in its infancy [18]. Evidence demonstrating that central components of the induced resistance circuitry, including the master regulatory protein NPR1, are conserved in rice has only recently been presented [19-22]. Moreover, reports on SAR-like phenomena in rice are scarce. Most tellingly in this regard, a 17-year-old report of systemically enhanced resistance against the rice blast pathogen *M. oryzae *triggered by a localized infection with the non-rice pathogen *P. syringae *pv. *syringae *remains one of the most compelling examples of a SAR-like response in rice to date [23]. In contrast, there is a sizeable body of evidence demonstrating systemic protection against various rice pathogens resulting from ISR elicited by, amongst others, *Pseudomonas *[24,25], *Bacillus *[26] and *Serratia *strains [27]. However, in most if not all cases, still very little is known about the basic mechanisms governing this ISR response.

In a previous report, we demonstrated that rice plants of which the roots were colonized by the fluorescent pseudomonad *P. aeruginosa *7NSK2 developed an enhanced defensive capacity against infection with *M. oryzae*. Bacterial mutant analysis revealed that this 7NSK2-mediated ISR is based on secretion of the redox-active pigment pyocyanin. Perception of pyocyanin by the plant roots was shown to cue the formation of reiterative micro-oxidative bursts in naïve leaves, thereby priming these leaves for accelerated expression of HR-like cell death upon pathogen attack [28]. Aiming to gain further insight into the molecular mechanisms underpinning rhizobacteria-modulated ISR in rice, we tested the ability of the biocontrol agent *Serratia plymuthica *IC1270 to induce systemic resistance against various rice pathogens with different modes of infection. Originally isolated from the rhizosphere of grapes,*S. plymuthica *IC1270 is a well-characterized PGPR strain producing a broad palette of antimicrobial compounds [29-32]. In addition to its potential as a direct antagonist of a wide array of plant pathogens, preliminary experiments in bean and tomato revealed that IC1270 is equally capable of reducing disease through activation of a plant-mediated defense response [32]. Here, we demonstrate that colonization of rice roots by IC1270 renders foliar tissues more resistant to *M. oryzae*. Using a combined cytological and pharmacological approach, evidence is provided that IC1270 locks plants into a pathogen-inducible program of boosted ROS formation, culminating in the prompt execution of HR cell death at sites of attempted pathogen entry. Similar, yet even more pronounced, phenotypes of hypersensitively dying cells in the vicinity of fungal hyphae were observed in a genetically incompatible rice-*M. oryzae *interaction, suggesting that IC1270-mediated ISR and *R*-gene-mediated ETI involve similar defense mechanisms. However, this IC1270-inducible ISR seems to play an ambivalent role within the rice disease resistance network, as bacteria-treated plants were rendered hypersusceptible to the necrotrophic pathogens *R. solani *and *C. miyabeanus*

## Methods

### Cultivation of rhizobacteria and pathogens

Bacterial strains used in this study were *Serratia plymuthica *IC1270, which was originally described as *Enterobacter agglomerans *[29], and *Pseudomonas aeruginosa *7NSK2 [33]. For inoculation experiments, IC1270 and 7NSK2 were grown on iron-limiting King's B medium [KB; [34]] for 24 h at 28°C and 37°C, respectively. Bacterial cells were scraped off the plates and suspended in sterile saline (0.85% NaCl). Densities of the bacterial suspensions were adjusted to the desired concentration based on their optical density at 620 nm.

*Magnaporthe oryzae *isolate VT7, a field isolate from rice in Vietnam [35], was grown at 28°C on half-strength oatmeal agar (Difco, Sparks, USA). Seven-day-old mycelium was flattened onto the medium using a sterile spoon and exposed to blue light (combination of Philips TLD 18W/08 and Philips TLD 18W/33) for seven days to induce sporulation. Conidia were harvested as described in De Vleesschauwer *et al. *[28], and inoculum concentration was adjusted to a final density of 1 × 10^4 ^spores ml^-1 ^in 0.5% gelatin (type B from Bovine skin; Sigma-Aldrich G-6650).

*Rhizoctonia solani *isolate MAN-86, belonging to anastomosis group AG-1 IA [36], was maintained on potato dextrose agar (PDA; Difco Laboratories, Detroit, USA). Inoculum was obtained according to Rodrigues *et al. *[37] with minor modifications. After autoclaving, 15 toothpicks, 1 cm in length, and five agar plugs (5 mm in diameter), obtained from the margin of an actively growing colony of *R. solani*, were transferred to PDA plates. These plates were then incubated for 8 days at 28°C so *R. solani *could colonize the toothpicks.

*Cochliobolus miyabeanus *strain 988, obtained from diseased rice in field plots at the International Rice Research Institute (Manila, The Philippines), was grown for sporulation at 28°C on PDA. Seven-day-old mycelium was flattened onto the medium using a sterile spoon and exposed to blue light for three days under the same conditions mentioned above. Upon sporulation, conidia were harvested exactly as stated in Thuan *et al. *[35] and re-suspended in 0.5% gelatin to a final density of 1 × 10^4 ^conidia ml^-1^.

### Pathogen inoculation and disease rating

Four-week-old rice seedlings (5-leaf stage) were challenge-inoculated with *Magnaporthe oryzae *as described in De Vleesschauwer *et al. *[28]. Six days after inoculation, disease severity on the fourth leaves of each plant was rated by counting the number of elliptical to round-shaped lesions with a sporulating gray center, and expressed relative to non-induced control plants.

*R. solani *bioassays were performed essentially as described in Rodrigues *et al. *[37]. Plants were challenged when four weeks old by placing a 1-cm toothpick colonized by *R. solani *inside the sheath of the second youngest fully expanded leaf. Inoculated plants were maintained inside humid inoculation chambers (≥ 92% relative humidity; 30 ± 4°C) for 72 h, and, thereafter, transferred to greenhouse conditions. Four days after challenge infection, disease severity was assessed by measuring the length of the water-soaked lesions.

*C. miyabeanus *bioassays were performed as described in Ahn *et al. *[38] with minor modifications. Five-week-old seedlings (6.5-leaf stage) were misted with a *C. miyabeanus *spore suspension containing 1 × 10^4 ^conidia ml^-1 ^in 0.5% gelatin. Inoculated plants were kept in a dew chamber (≥ 92% relative humidity; 30 ± 4°C) for 18 h to facilitate fungal penetration, and subsequently transferred to greenhouse conditions for disease development. Disease symptoms were scored at four days after inoculation for about 48 leaves per treatment. Disease ratings were expressed on the basis of diseased leaf area and lesion type: I, no infection or less than 2% of leaf area infected with small brown specs less than 1 mm in diameter; II, less than 10% of leaf area infected with brown spot lesions with gray to white center, about 1–3 mm in diameter; III, average of about 25% of leaf area infected with brown spot lesions with gray to white center, about 1–3 mm in diameter; IV, average of about 50% of leaf area infected with typical spindle-shaped lesions, 3 mm or longer with necrotic gray center and water-soaked or reddish brown margins, little or no coalescence of lesions; V, more than 75% of leaf area infected with coalescing spindle-shaped lesions.

### Induction treatments

Induced systemic resistance (ISR) assays were performed as described in De Vleesschauwer *et al. *[28] with minor modifications. Briefly, rice plants (*Oryza sativa *spp. *indica *line CO39) were grown under greenhouse conditions (30 ± 4°C, 16-h photoperiod) in commercial potting soil (Structural; Snebbout, Kaprijke, Belgium) that had been autoclaved twice on alternate days for 21 min. Rice seeds first were surface sterilized with 1% sodium hypochlorite for two min, rinsed three times with sterile, demineralized water and incubated for five days on a wet sterile filter paper in sealed Petri dishes at 28°C. Prior to sowing in perforated plastic trays (23 by 16 by 6 cm), roots of germinated seeds were dipped in a bacterial suspension of the ISR-inducing strains [5 × 10^7 ^colony-forming units (cfu) ml^-1^] for 10 min. The autoclaved soil was thoroughly mixed with bacterial inoculum to a final density of 5 × 10^7 ^cfu ml^-1^. To ensure consistent root colonization by the eliciting bacteria, rice plants were soil-drenched a second time with bacterial inoculum (5 × 10^7 ^cfu ml^-1^) at ten days after sowing. In control treatments, soil and rice plants were treated with equal volumes of sterilized saline.

For experiments in which purified pyocyanin was applied to the roots of rice seedlings, plants were grown in a hydroponic gnotobiotic system as described before [28]. In this system, plants were fed with various concentrations of pyocyanin and ascorbate 4 days before challenge inoculation by adding the desired concentration to the half-strength Hoagland nutrient solution. Pyocyanin extraction, quantification and application were performed exactly as stated in De Vleesschauwer et al. [28].

### Evaluation of plant colonization by *S. plymuthica *IC1270 and *P. aeruginosa *7NSK2

Bacterial colonization of the plant roots was determined by the time the bioassays were discontinued. Roots of three plants of each treatment were rinsed to remove most of the soil, weighed, and 1 g of root was macerated in sterile demineralized water. Serial dilutions were plated on KB agar supplemented with rifampicin (40 μg/ml) for IC1270, and KB agar for 7NSK2. After overnight incubation at 28°C and 37°C for IC1270- and 7NSK2-treated roots, respectively, the number of colony-forming units per gram of root fresh weight was determined. Possible spreading of root-inoculated bacteria to distal leaves was checked as described before [28]. The detection limit of this assay is approximately 10 CFU per sheath or leaf blade.

### Cytological analysis of IC1270-mediated ISR against *M. oryzae*

To gain more insight into the nature of IC1270-mediated ISR against *M. oryzae*, cytological studies were performed at sites of pathogen entry. To this purpose, we adopted the intact leaf sheath assay previously described by Koga *et al. *[39]. Briefly, leaf sheaths of the fifth leaf of rice plants at the 5.5 leaf stage were peeled off with leaf blades and roots. The leaf sheath was laid horizontally on a support in plastic trays containing wet filter paper, and the hollow space enclosed by the sides of the leaf sheaths above the mid vein was filled with a suspension of spores (5 × 10^4 ^conidia ml^-1^) of *M. oryzae*. Inoculated leaf sheaths were then incubated at 25°C with a 16-h photoperiod. When ready for microscopy, the sheaths were hand-trimmed to remove the sides and expose the epidermal layer above the mid vein. Lower mid vein cells were removed to produce sections three to four cell layers thick. At least five trimmed sheath tissue sections originating from different control and IC1270-treated plants were used for each sampling point.

Phenolic compounds were visualized as autofluorescence under blue light epifluorescence (Olympus U-MWB2 GPF filter set-excitation: 450 to 480 nm, dichroic beamsplitter; 500 nm, barrier filter BA515). To detect H_2_O_2 _accumulation, staining was performed according to the protocol of Thordal-Christensen *et al. *[40] with minor modifications. Six hours before each time point, trimmed sheath segments were vacuum-infiltrated with an aqueous solution of 1 mg ml^-1 ^3,3'-diaminobenzidine(DAB)-HCL (pH = 3.8) for 30 min. Thereafter, infiltrated segments were incubated in fresh DAB solution until sampling. DAB polymerizes in the presence of H_2_O_2 _and endogenous peroxidase to form a brownish-red precipitate that can be easily visualized using bright-field microscopy. After staining, trimmed sheath segments were mounted in 50% glycerol. Images were acquired digitally (Olympus Color View II camera, Aartselaar, Belgium) and further processed with the Olympus analySIS cell^F software.

### Artificial manipulation of the oxidative burst in detached rice leaves

For experiments in which plants were treated with the ROS-generating mixtures glucose plus glucose oxidase (G/GO) and xanthine plus xanthine oxidase (X/XO), fifth-stage leaves of four-week-old rice plants were excised and cut into 7-cm segments. *Aspergillus niger *glucose oxidase (Sigma-Aldrich, St. Louis, MO) was added to 2 mM D-glucose in 20 mM Na phosphate buffer, pH 6.5, immediately prior to plant treatment (100 units ml^-1^). Similarly, xanthine oxidase (0.1 units ml^-1^) was added to 1 mM xanthine in the same buffer solution (Sigma-Aldrich, St. Louis, MO). The ROS-generating mixtures, buffer alone or buffer containing glucose (2 mM), gluconate (50 μM), glucose oxidase (100 units ml^-1^), xanthine (1 mM), or xanthine oxidase (0.1 units ml^-1^) were infiltrated in approximately 20 μl aliquots into five sites on the abaxial surface of the detached leaf segments using a syringe without a needle. Alternatively, detached leaf segments were infiltrated with 3-aminotriazole (10 mM) or catalase (1100 units ml^-1^) in 10 mM MES buffer, pH 6.5. *In planta *H_2_O_2 _generation by G/GO, X/XO, or 3-aminotriazole was visually confirmed by means of abovementioned DAB staining procedure. Upon infiltration, detached leaf segments were immediately placed onto a glass slide in 14.5 × 14.5 cm Petri dishes lined with moist filter paper. Two hours later, 10 μl of *M. oryzae *or *C. miyabeanus *conidial suspension (5 × 10^4 ^sp ml^-1 ^in 0.25% gelatin) was drop-inoculated in the center of the infiltrated regions. Control leaves were mock-inoculated with a 0.25% (wt vol^-1^) gelatin suspension. After 24 h, the droplets were removed with a laboratory tissue. For challenge with *R. solani*, a 0.8-cm-diameter mycelial disc of a 7-day-old PDA culture of *R. solani *strain MAN-86 was carefully placed in the center of the infiltrated region. As a control, leaf segments were inoculated with a PDA plug without hyphae. Petri dishes with inoculated leaf segments were routinely placed on a laboratory bench and maintained at 21°C to 26°C with a 16 h photoperiod. For *M. oryzae *and *C. miyabeanus *assays, disease development was assessed 96 h post-inoculation using digital image analysis (APS assess software; Lakhdar Lamari, Winnipeg, Canada) for quantification of necrotic leaf areas. These areas were represented as the number of pixels and expressed as a percentage of the total pixel number in a fixed 1 cm^2^-leaf quadrant. In case of *R. solani *inoculation, disease ratings were visually graded into five classes based on the leaf area affected; 1 = no infection, 2 = 1 to 10%, 3 = 11 to 25 %, 4 = 26 to 50%, and 5 = more than 50% of leaf area affected.

## Results

### Differential effectiveness of ISR triggered by *S. plymuthica *IC1270

To assess the ISR-triggering capacity of *S. plymuthica *IC1270, susceptible rice plants were grown in soil containing IC1270 bacteria, and subsequently challenged with several fungal pathogens exhibiting different modes of infection. In these ISR bioassays, the resistance-inducing potential of IC1270 was compared to that of *P. aeruginosa *7NSK2, a well-studied PGPR strain which we previously uncovered as a potent activator of induced resistance responses in rice [28].

**Figure 1:**
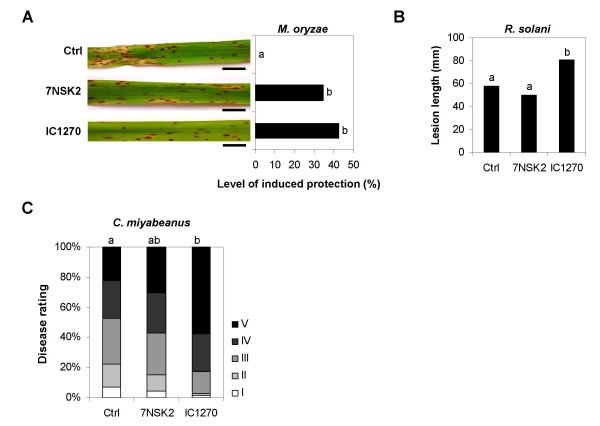
**Spectrum of effectiveness of *Pseudomonas aeruginosa *7NSK2- and *Serratia plymuthica *IC1270-triggered ISR in rice**. ISR was induced by growing the plants in soil containing 7NSK2 or IC1270 bacteria. Control plants were treated with water. (**A**) Quantification of ISR against *M. oryzae*. Plants were challenged when 4 weeks old by spraying a spore suspension of virulent *M. oryzae *VT7. Six days after challenge inoculation, disease was quantified by counting the number of susceptible-type lesions per leaf 4 and the level of induced protection was calculated relative to challenged control plants. Photographs depicting representative symptoms were taken 7 days post inoculation. Bar = 10 mm. (**B**) Quantification of ISR against *R. solani*. Four-week-old plants were challenged by placing a 1 cm-toothpick colonized by *R. solani *inside the sheath of the second youngest fully developed leaf; 4 days later, disease severity was assessed by measuring the total length of sheath blight lesions. (**C**) Quantification of ISR against *C. miyabeanus*. Plants were challenge-inoculated when five weeks old by spraying a conidial suspension. Disease evaluation was performed 4 d postinoculation, using a 1-to-5 disease severity scale as described in the Methods section. For all graphs, statistical analysis was performed on pooled data from at least four independent experiments, because interaction between treatment and experiment was not significant at α = 0.05 and variances were homogeneous. Different letters indicate statistically significant differences between treatments according to non-parametric Kruskall-Wallis and Mann-Whitney tests (n ≥ 42; α = 0.05).

We first tested whether root colonization by *S. plymuthica *IC1270 exerts a protective effect against infection by the hemibiotrophic ascomycete *M. oryzae*, causal agent of the devastating rice blast disease and a major threat to food security worldwide [41]. By 4 days post-inoculation (dpi), leaves of control, non-induced plants displayed typical water-soaked, diamond-shaped lesions, developing conidia at the center of each lesion by 6 dpi. In contrast, IC1270-colonized plants exhibited a marked reduction in the number of these susceptible-type lesions, producing a resistance phenotype mimicking that of quantitative trait loci-governed intermediate resistance (Fig. 1A). This resistance type is characterized by the abundance of small necrotic non-sporulating lesions, less than 2 mm in diameter, 60 to 72 h post-inoculation (hpi). Consistent with our previous findings [28], treatment with *P. aeruginosa *7NSK2 resulted in a substantial reduction of disease as well. No significant differences in the number of susceptible-type lesions could be observed between IC1270- and 7NSK2-treated plants, indicating that IC1270 and 7NSK2 are equally effective in suppressing *M. oryzae*.

Because IC1270 clearly inhibited the growth of *M. oryzae *in dual culture experiments (data not shown), possible systemic plant colonization by the rhizobacteria was checked. However, in all bioassays performed, IC1270 bacteria were absent from sheaths or leaves of root-induced plants, indicating that bacterial colonization remained confined to the root zone (data not shown). Although such spatial separation does not rule out the possibility that IC1270-conferred protection might result from long-distance translocation of bacteria-produced allelochemicals to systemic leaves, the latter is rather unlikely as pilot experiments aimed at elucidating the bacterial traits underpinning IC1270-ISR revealed that mutants defective in the global response regulator protein GacA [30], which controls the synthesis of various antifungal metabolites (e.g. chitinases and pyrrolnitrin), were as effective as wild-type IC1270 in reducing rice blast disease severity (De Vleesschauwer and Höfte, unpublished results). The cumulative data therefore strongly suggest that the beneficial protective activity exerted by *S. plymuthica *IC1270 is based on activation of the plant's defensive repertoire, rather then being caused by microbial antagonism.

To test the spectrum of effectiveness of this IC1270-mediated ISR, we next assayed for induction of resistance against the sheath blight pathogen, *Rhizoctonia solani*, and the brown spot pathogen, *Cochliobolus miyabeanus*, both of which are considered necrotrophic fungi. In contrast to *M. oryzae*, which sequentially invades living cells [42], *R. solani *and *B. oryzae *kill host cells at very early stages in the infection, leading to extensive tissue damage [43]. As shown in Fig 1B, both IC1270 and 7NSK2 failed to reduce disease caused by *R. solani*. This impaired ISR response was not due to insufficient root colonization as bacterial counts in the rhizosphere of treated rice seedlings were comparable to those obtained in the *M. oryzae *bioassays (1.14 ± 0.19 × 10^5 ^CFU. g^-1^). Interestingly, in all four independent experiments, IC1270 pretreatment favored subsequent infection by *R. solani*, causing an average 39.6% increase in disease severity relative to non-induced controls. A similar trend was observed when challenging with *C. miyabeanus*, with IC1270 consistently promoting vulnerability to the latter pathogen (Fig. 1C). Root colonization by 7NSK2, however, yielded variable results. No significant differences between control and 7NSK2-treated plants could be observed in three bioassays, whereas in the two remaining assays, root treatment with 7NSK2 rendered rice seedlings substantially more susceptible to brown spot.

In all experiments, mock-inoculated control plants remained healthy, and no apparent differences in appearance, size, or weight of control, 7NSK2 or IC1270-treated plants were observed prior to challenge infection (data not shown). Thus, under the experimental conditions used in this study, root treatment with the ISR-inducing bacteria did not lead to detectable effects on plant growth that could have affected the growth or development of the respective pathogens.

Collectively, these findings demonstrate that *S. plymuthica *IC1270 plays an ambivalent role in the rice induced resistance network, acting as a potent elicitor of resistance to the hemibiotroph *M. oryzae *while promoting susceptibility to the necrotrophs *C. miyabeanus *and *R. solani*.

### *S. plymuthica *IC1270 triggers HR-like responses at the sites of pathogen attack

To begin to unravel the defense mechanism(s) underpinning IC1270-mediated ISR, we analyzed the cytological alterations associated with restriction of *M. oryzae *in IC1270-induced plants using the intact leaf sheath method designed by Koga and associates [39]. In this system, intact leaf sheaths of control, non-induced and IC1270-treated plants of the highly susceptible rice variety CO39 were routinely inoculated by injecting a conidial suspension of the virulent blast isolate VT7. For comparison with *R *gene-mediated ETI, we also included the VT7-resistant variety C101LAC, the latter being a near-isogenic line of CO39 carrying the blast resistance genes *Pi-1 *and *Pi-33 *[44,45].

**Figure 2:**
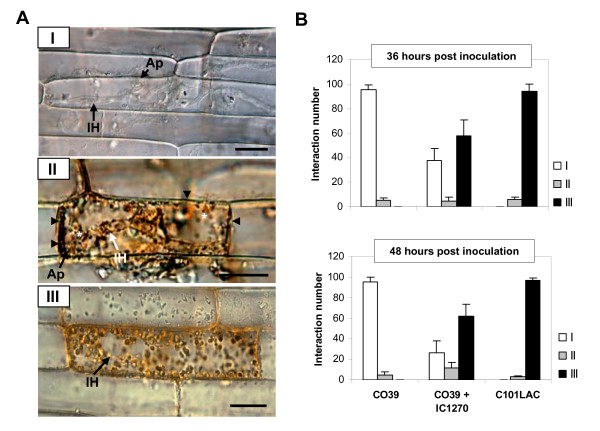
**Influence of root treatment with *S. plymuthica *IC1270 on *M. oryzae*-induced cellular responses in rice**. (**A**) Intact leaf sheaths of the susceptible cv. CO39 and its resistant near-isogenic line C101LAC were challenged by injecting a conidial suspension of *M. oryzae *VT7. Left, Micrographs depicting representative interaction phenotypes (48 hpi): (I), Vigorous invasion of living tissues in the absence of visible host responses (CO39; Control treatment). (II), Fungal arrest in the first-invaded cell associated with browning of anticlinal cell walls (black arrowheads) and enhanced vesicular activity (asterisks) [CO39; Control treatment]. (III), Abrupt arrest of fungal invasion in hypersensitively reacting epidermal cell as indicated by dense cytoplasmic aggregation (C101LAC; Control treatment). Ap, appressorium or appressorial site. IH = invading hyphae. Scale bars = 20 μm. (**B**) Frequencies of abovementioned interaction phenotypes at 36 and 48 hours post inoculation. Each bar represents the mean and SD of six replications stemming from three plants. At least 50 single-cell interaction sites originating from representative sheath sections were examined per replication. Data from one experiment is presented. Repetition of experiments led to results very similar to those shown.

No obvious alterations in cell physiology due to IC1270 treatment were observed prior to infection. Similarly, quantitative recording of attempted blast infections revealed no significant differences in the number of unsuccessful penetration events, indicating that both IC1270-mediated ISR and *R*-gene-conditioned ETI are unlikely to impede pre-penetration development by *M. oryzae *(data not shown). On the other hand, epidermal cells were found to respond to fungal ingress through various cellular reaction types depicted at 48 hpi in Fig 2A. A susceptible reaction was manifested as a type 1 phenotype in which extensively branched invasive hyphae vigorously invaded living epidermal cells with little or no **visible **host response. Interaction phenotype 2, on the other hand, was characterized by prompt arrest of fungal growth in the first-invaded epidermal cell, a phenomenon associated with enhanced vesicular activity and browning of the anticlinal cell walls, while a type 3 reaction represented infection sites in which fungal invasion was curtailed shortly after penetration due to development of HR-like cell death, as indicated by the characteristic aggregation of the cytoplasm and a bright autofluorescence of the anticlinal cell walls [39,46]. As expected, sheath cells of non-induced, susceptible CO39 plants inoculated with virulent VT7 predominantly mounted a type 1 reaction, whereas HR was the prevailing plant response in the incompatible interaction between VT7 and C101LAC. Most conspicuously, IC1270-induced CO39 sheath cells displayed an interaction profile resembling that observed in VT7-invaded sheaths of genetically resistant C101LAC, with type 3 reactions accounting for approximately 60% of all interactions by 48 hpi (Fig. 2B).

At later stages of infection, *M. oryzae *had massively colonized the epidermis and mesophyll of CO39 sheaths causing extensive host damage as evidenced by the ubiquitous presence of cellular debris and fragmented remnants of host cell walls around invasive hyphae in the mesophyll (data not shown). By contrast, in resistant C101LAC, as well as in IC1270-induced CO39, invading hyphae were largely trapped within hypersensitively dying cells in the epidermal layer, preventing fungal passage to the underlying tissue.

**Figure 3:**
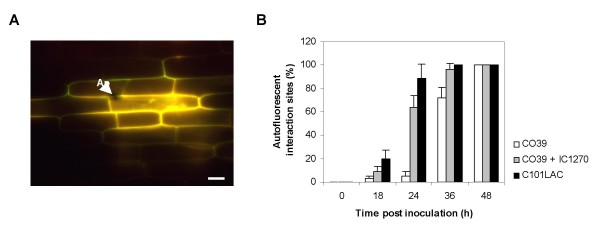
***S. plymuthica *IC1270 primes rice for enhanced accumulation of autofluorescent phenolics upon challenge inoculation**. Intact leaf sheaths of the susceptible cv. CO39 and its resistant near-isogenic line C101LAC were challenged by injecting a conidial suspension of *M. oryzae *VT7. (**A**) Epifluorescence image of IC1270-induced sheath cells at 24 hpi. Ap = appressorium. Scale bar = 20 μm. (**B**) Quantitative analysis of autofluorescence under blue light excitation in water-treated susceptible (CO39), susceptible yet ISR-expressing (CO39 + IC1270), and genetically resistant (C101LAC) plants. Each bar represents the mean and SD of six replications stemming from three plants. At least 50 single-cell interaction sites originating from representative sheath sections were examined per replication. Data from one experiment is presented. Repetition of experiments led to results very similar to those shown.

Because rapid accumulation of phenolic compounds is a hallmark of rice defense against *M. oryzae *[46,47], we also examined the effect of IC1270 pre-treatment on the level of autofluorescence. Autofluorescence was detectable as early as 18 hpi, irrespective of IC1270 treatment or the level of resistance of the cultivars used (Fig. 3A). However, similar to what was observed in resistant C101LAC, root treatment of CO39 with IC1270 caused the frequency of autofluorescent appressorial sites to increase rapidly from 18 hpi onward, reaching a level of 60 and 100% of all interactions by 24 and 36 hpi, respectively (Fig. 3B). By contrast, in non-induced CO39 cells, less than 6% of the appressorial sites showed autofluorescence 24 hpi, indicating that root colonization by IC1270 primes rice sheath cells for accelerated deposition of autofluorescent phenolic compounds at sites of attempted pathogen invasion. Along with the high frequency of hypersensitively reacting cells, these observations suggest that IC1270-mediated ISR and *R*-gene-conditioned ETI act, at least in part, through a similar set of defense reactions.

### *S. plymuthica *IC1270-mediated ISR to *M. oryzae *involves priming for enhanced attacker-induced H_2_O_2 _generation

**Figure 4:**
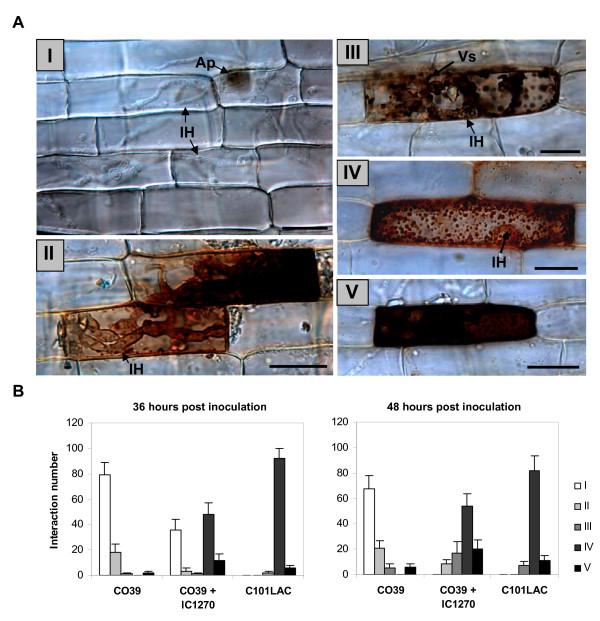
**Influence of treatment with *S. plymuthica *IC1270 on *M. oryzae*-induced H_2_O_2_-generation in epidermal sheath cells**. Intact leaf sheaths of the susceptible rice cv. CO39 and its resistant near-isogenic line C101LAC were challenged by injecting a conidial suspension of *M. oryzae *VT7. (**A**), Micrographs depicting distinct H_2_O_2 _accumulation patterns at 48 hpi in inoculated leaf sheaths supplied with 3,3'-diaminobenzidine (DAB): (I), successful fungal colonization of living epidermal cells in the absence of DAB staining (CO39; Control treatment); (II) DAB accumulation in the first-invaded cell following fungal invasion of adjacent cells (CO39; IC1270 treatment); (III) accumulation of DAB-positive vesicle-like bodies in the vicinity of the invasive hyphae (CO39; IC1270 treatment); (IV) DAB-positive cytoplasmic granules in hypersensitively reacting cells (C101LAC; Control treatment); (V) whole-cell DAB staining (CO39; Control treatment). Ap, appressorium or appressorial site; IH, invading hyphae; Vs, vesicles. Scale bars = 20 μm. (**B**), Frequencies of abovementioned DAB patterns at 36 and 48 hours post inoculation. In all graphs, bars represent the mean and SD of six replications originating from three plants. At least 50 single-cell interaction sites originating from representative sheath sections were examined per replication. Data from one experiment is presented. Repetition of experiments led to results very similar to those shown.

There is ample evidence demonstrating the active involvement of reactive oxygen species (ROS), and H_2_O_2 _in particular, in the induction, signaling and execution of blast resistance in rice [48-51]. Furthermore, in the course of previous studies, we demonstrated that pyocyanin-induced H_2_O_2 _microbursts are primordial for the onset of *P. aeruginosa *7NSK2-mediated ISR against *M. oryzae *[28]. Taking these facts into account, we sought to extend our cytological analysis of ISR elicited by IC1270 by monitoring the spatiotemporal patterns of pathogenesis-related H_2_O_2 _production. *In planta *accumulation of H_2_O_2 _was visualized using an endogenous peroxidase-dependent staining procedure with 3,3'-diaminobenzidine (DAB). In these DAB assays, reddish-brown precipitates are deposited at the sites of H_2_O_2 _accumulation [40]. No DAB accumulation was observed in mock-inoculated controls, regardless of IC1270 treatment or the inherent level of resistance of the cultivars used. However, comparative analysis of H_2_O_2 _production in pathogen-inoculated seedlings revealed the occurrence of a wide range of distinct DAB staining patterns that could be grouped into five categories (Fig. 4A). The first type comprised interaction sites in which DAB accumulation was not detectable despite massive fungal colonization of both penetrated and neighboring epidermal cells. Conversely, interaction sites displaying H_2_O_2 _accumulation in the primary invaded epidermal cell following spread of the invasive hyphae into neighboring cells were classified as a type II reaction. Type III interaction sites were characterized by the ubiquitous occurrence of DAB-positive vesicle-like bodies targeted to the invading hyphae. A type IV reaction referred to intracellular DAB staining tightly associated with the characteristic cytoplasmic aggregates of HR-expressing cells (type IV), while interaction sites displaying whole-cell DAB accumulation were scored as a type V reaction. Importantly, when the DAB solution was supplemented with ascorbate, staining was abolished, indicating that the staining was due to H_2_O_2 _(data not shown).

Leaf sheath cells of susceptible CO39 were characterized by the high ratio of H_2_O_2_-negative type I reactions, accounting for 78% and 67% of all interaction sites by 36 and 48 hpi, respectively (Fig. 4B). In some incidences (21% of all interaction sites at 48 hpi), H_2_O_2_accumulated in the initially penetrated epidermal cell following the formation of an extensively branched mycelium in the neighboring cells. Yet, this type II reaction seemingly occurred too late to effectively stall the pathogen. IC1270-induced CO39 cells, on the other hand, exhibited a strikingly different set of responses in that type I reactions, reaching a level of 33% at 36 hpi, were no longer discernible by 48 hpi. The rapid decline in the frequency of type I reactions from 36 hpi onward corresponded to an approximately 15% increase in the frequency of both type III and type V reactions. HR-like cell death of attacked epidermal cells, seen at approximately 52% of all interaction sites, was always associated with H_2_O_2 _accumulation in the cytoplasmic aggregates, beginning 32 hpi. Although not identical, by 48 hpi the H_2_O_2 _signature of IC1270-treated CO39 plants showed substantial similarity to that observed in the incompatible interaction between C101LAC and VT7, thereby further emphasizing the possible mechanistic similarities between IC1270-mediated ISR and R-protein-dictated ETI.

Starting 50 hpi, a strong accumulation of H_2_O_2 _was found in CO39 mesophyll cells that appeared to collapse, whereas in samples from IC1270-induced CO39 or C101LAC sheaths, DAB staining in the mesophyll layer was seldom observed (data not shown). However, at these late infection stages, massive H_2_O_2 _accumulation is most likely a consequence of progressive cellular destruction and overtaxed anti-oxidative capacities, and hence, a chaotic reaction associated with susceptibility, rather than a controlled defense response restricting cellular accessibility for *M. oryzae*. Together these results clearly demonstrate the potential of IC1270 to prime rice for augmented generation of epidermis-localized H_2_O_2_.

### Manipulation of oxidative stress in inoculated leaves

**Figure 5:**
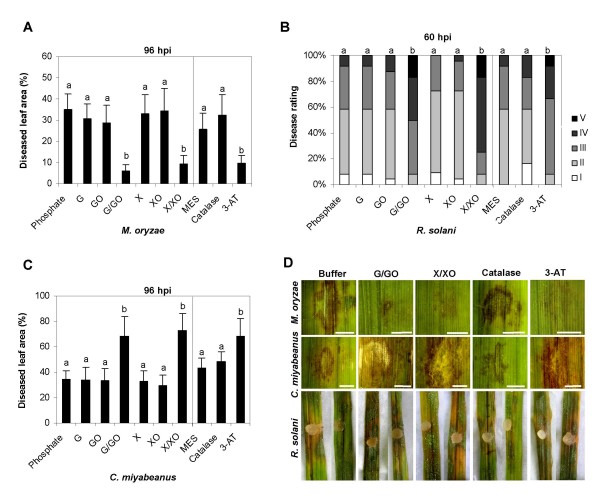
**Effect of artificial ROS manipulation on *M. oryzae*, *C. miyabeanus *and *R. solani *infection**. For continuous generation of H_2_O_2 _*in situ*, detached leaves were infiltrated with mixtures of glucose oxidase (GO; 100 units ml^-1^) plus glucose (G; 2 mM), or xanthine oxidase (XO; 0.1 units ml^-1^) plus xanthine (X; 1 mM). Control plants were treated with buffer solution only (50 mM phosphate, pH = 6.5). Alternatively, plants were infiltrated with 3-aminotriazole (3-AT; 10 mM) or catalase (CAT; 1100 units ml^-1^) with MES buffer-treated plants as corresponding controls. Two hours later, 10 μl droplets of conidial suspension of *M. oryzae *or *C. miyabeanus *were carefully applied to the center of the infiltrated area. For infection with *R. solani*, 8-mm mycelium-overgrown agar plugs were used. After 4 days of incubation under laboratory conditions, *M. oryzae *and *C. miyabeanus *symptom development was assessed using digital image analysis for quantification of necrotic leaf areas. The intensity of the *R. solani *symptoms was evaluated 60 h post-inoculation and graded into five categories based on the leaf area affected as described in the Methods section. In all graphs, bars represent the mean and SD of twenty-four leaf segments. Different letters indicate statistically significant differences between treatments (*M. oryzae *and *C. miyabeanus*, Fisher's LSD test, α = 0.05; *R. solani*, Mann-Whitney, α = 0.05). Photographs depicting representative symptoms were taken 96 hpi in case of *M. oryzae *and *C. miyabeanus *challenge, and 60 hpi in case of challenge with *R. solani*.

In light of the well-documented ability of ROS to serve multiple defense-related signaling functions, sometimes with opposite effects in different contexts [52,53], we asked whether the ability of IC1270 to boost pathogenesis-related H_2_O_2 _generation might account for the differential effectiveness of IC1270-mediated ISR against *M. oryzae*, *R. solani *and *C. miyabeanus*. To address this question, we examined the effect of manipulating the oxidative stress in pathogen-inoculated leaves on subsequent disease development. To artificially raise the level of ROS in inoculated leaves, detached leaves were pressure-infiltrated with mixtures of glucose plus glucose oxidase (G/GO) and xanthine plus xanthine oxidase (X/XO). Similar to what has been observed in other plant species [54,55], supplying rice leaves with G/GO resulted in the sustained production of H_2_O_2_within the apoplast (see Additional file 1), whereas a mixture of xanthine and xanthine oxidase was found to generate both superoxide and H_2_O_2_, the latter by dismutation (data not shown). Treatment with either compound (i.e. xanthine or glucose) or with the enzymes alone had no significant effect on disease development compared to buffer-treated control leaves (Figs 5A, B, C). However, infiltration of G/GO or X/XO dramatically reduced the size of the necrotic lesions incited by *M. oryzae *infection (Figs. 5A, D). By contrast, pre-treatment with G/GO or X/XO mixtures strongly stimulated necrosis induced by *R. solani *(Fig. 5B). By 60 hours after infection, the majority of ROS-treated and *Rhizoctonia*-inoculated leaves showed extensive necrosis and were almost completely deteriorated (Fig. 5D). Enhanced ROS generation also greatly enhanced lesion formation by *C. miyabeanus*, suggesting a common pathogenicity mechanism for both these necrotrophs (Figs. 5C, D). Extensive lesions were also observed when manipulating plant-intrinsic catalase activity. Although exogenous catalase did not significantly alter lesion development, infiltration of rice leaves with a specific catalase inhibitor, 3-aminotriazole, prior to inoculation, was indistinguishable from the G/GO- or X/XO-treated leaves. No lesions were detected in leaves infiltrated with ROS-producing mixtures, catalase or 3-AT alone, as previously reported [56].

**Figure 6:**
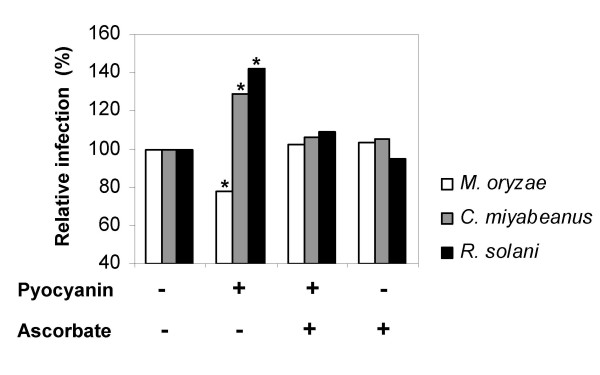
**Effect of ascorbate on resistance to *M. oryzae*, *C. miyabeanus *and *R. solani *in pyocyanin-amended hydroponically-grown rice plants**. Purified pyocyanin (100 nM) and/or ascorbate (50 μM) were added to the half-strength Hoagland nutrient solution 4 days prior to challenge inoculation. For details on *M. oryzae*, *C. miyabeanus *and *R. solani bioassays*, see legend to Fig 1. The values presented are from representative experiments that were repeated three times with similar results. Asterisks indicate statistically significant differences with the non-treated control (Kruskall-Wallis and Mann-Whitney, α = 0.05, n = 24).

Building on our earlier work with respect to 7NSK2-mediated ISR, we sought to extend our analysis of the proposed dual role of ROS in rice defense by feeding the pro-oxidative pigment pyocyanin to hydroponically grown rice plants and observe any effects on plant resistance. Opposite to the enhanced resistance observed against *M. oryzae*, pyocyanin feeding favored subsequent infection by both *C. miyabeanus *and *R. solani *(Fig. 6). Amending the pyocyanin solution with ascorbate, which has long been recognized as a major antioxidant buffer and free-radical scavenger [57], severely attenuated the pyocyanin-provoked resistance or susceptibility, corroborating our previous findings [28]. Taken together, these results clearly demonstrate that enhanced ROS levels in inoculated leaves positively influence resistance to *M. oryzae*, while exerting a negative effect on rice defense to *C. miyabeanus *and *R. solani*.

## Discussion

Despite the emergence of rice as a pivotal model for molecular genetic studies of disease resistance in cereal crops, molecular information regarding chemically and biologically induced defenses is still largely missing. In an effort to broaden our understanding of the rice induced resistance machinery, we analyzed the host defense responses underpinning ISR triggered by the biocontrol agent *S. plymuthica *IC1270. The results presented in this study demonstrate that root colonization by IC1270 predisposes rice to undergo a massive oxidative burst and related HR-like cell death at sites of attempted pathogen invasion, a process culminating in heightened resistance to the hemibiotrophic blast pathogen, *M. oryzae*. The same treatment, however, rendered plants more susceptible to attack by the necrotrophic pathogens *R. solani *and *C. miyabeanus*. Besides tagging ROS and HR-like cell death as two-faced players in the rice defense response, these findings strengthen the argument that rice requires distinct mechanisms for defense against *M. oryzae *and the necrotrophs *R. solani *and *C. miyabeanus*.

Mounting evidence indicates that generation of systemic resistance does not necessarily require direct activation of defense mechanisms, but can also result from a faster and stronger activation of basal defenses in response to pathogen attack [17]. For instance, unlike pathogen-induced SAR, classic rhizobacteria-mediated ISR in Arabidopsis is not associated with a direct induction of defense mechanisms, but with priming for augmented defense activation upon challenge inoculation [58,59]. Other ISR-inducing PGPRs also have been found to enhance the plant's defensive capacity by hyper-activating pathogen-activated defenses [60-64], suggesting that priming for enhanced defense is a common mechanism in PGPR-mediated ISR. The results presented in this study add further support to this concept as root colonization by IC1270 did not cause a strong constitutive resistance phenotype, but rather primed plants to hyper-respond to subsequently inoculated pathogens, resulting in excessive defense activation and enhanced resistance to *M. oryzae*. This priming effect of IC1270 was borne out by the observation that challenge inoculation of IC1270-colonized plants with *M. oryzae *entailed a rapid accumulation of autofluorogenic phenolic compounds in and around epidermal cells displaying dense cytoplasmic granulation (Figs. 2 and 3), two features that are considered as hallmarks of an ETI-associated HR [46,47].

Comparative profiling of pathogenesis-related H_2_O_2 _accumulation in blast susceptible, yet ISR-expressing, and genetically resistant leaf sheath cells, further strengthened the parallels between R protein-mediated ETI and IC1270-triggered ISR priming (Fig. 4). Hence, IC1270 appears to protect rice from *M. oryzae *by reprogramming pathogen-attacked epidermal cells to undergo a rapid HR-like response, thereby providing a possible functional interface between rhizobacteria-mediated ISR and avirulent pathogen-induced ETI. Such mechanistic similarities between ISR and ETI are compatible with the idea that defense signals from multiple 'entry points' can converge and target overlapping sets of defense effectors [65-67]. Particularly relevant in this regard is the substantial overlap between gene expression changes and alterations in SA content induced during an avirulent pathogen-triggered ETI response, and those induced by treatment with flg22, an 22-amino-acid epitope of the archetypal MAMP elicitor flagellin [8,67,68]. Although unequivocal evidence is still lacking, the striking homologies with the sensitive perception mechanisms for pathogen-derived MAMPs that function in PTI suggest that ISR-triggering rhizobacteria are recognized in a similar manner [31,69]. In this perspective, it is not inconceivable that the mechanistic parallels between IC1270-mediated ISR and ETI can be traced to converging MAMP- and R-protein-induced defense responses. Furthermore, consistent with the view of ETI as an accelerated and amplified PTI response [1,70,71], such MAMP-orchestrated ISR elicitation may also explain the partial nature of the IC1270-induced resistance against *M. oryzae*.

Apart from *S. plymuthica *IC1270, several other biological and chemical agents have been shown to be capable of inducing resistance to *M. oryzae *[19,72], among which the SA analog BTH and the redox-active pigment pyocyanin, key determinant of ISR induced by *P. aeruginosa *7NSK2 [19,28]. Interestingly, both these resistance inducers appear to mimic IC1270 in that they produce a similar resistance phenotype, characterized by hypersensitively dying cells in the vicinity of fungal hyphae [28,38,73,74]. Although it does not follow that the signaling conduit(s) governing IC1270-mediated ISR is (are) necessarily the same as that (those) leading to pyocyanin- or BTH-inducible blast resistance, such commonalities apparent at the level of defense mobilization suggest that these elicitors may feed into related, if not identical, resistance pathways. Further supporting this hypothesis is the overlap manifest at the level of resistance to attackers, with IC1270, BTH and pyocyanin all being ineffective or even increasing vulnerability to *C. miyabeanus *and *R. solani *[28,38,75]. Intriguingly, induction of ISR by the PGPR strain *P. fluorescens *WCS374r appears to rely on a different resistance strategy and was found to be associated with priming for a diverse set of HR-independent cellular defenses, the prompt elaboration of invading hyphae-embedding tubules being a prominent component [76]. Considering this apparent plasticity in the molecular processes leading to induced resistance against *M. oryzae*, it is tempting to speculate that rice is endowed with multiple blast-effective induced resistance pathways.

The rapid production of ROS during the so-called oxidative burst is a hallmark of the plant's defense response. Although ROS are generally viewed as initiating agents in the disease resistance network [57], accumulating evidence indicates that ROS formation can cascade either to the detriment or benefit of the plant depending on the lifestyle and parasitic habits of the invading pathogen [5,10]. Hence, ROS can play a dual role in pathogen defense, acting as key players in resistance to biotrophic pathogens on the one hand [53,77], while weakening necrotroph resistance by assisting pathogen-induced host cell death on the other [5,56,78,79]. Taking these facts into account, we propose that priming for enhanced ROS generation may likewise function in IC1270-mediated ISR, thereby accounting for the differential effectiveness of this resistance against hemibiotrophic and necrotrophic pathogen assault. Critical to the formation of a hypothesis of primed ROS generation as a key event in ISR by IC1270 was the observation that artificially increased H_2_O_2 _levels, either resulting from infiltration of ROS-generating mixtures, inhibition of endogenous catalase activity or hydroponic feeding of pro-oxidative pyocyanin, faithfully mimicked IC1270 in conditioning resistance to *M. oryzae *but susceptibility to *C. miyabeanus *and *R. solani*. Although we are aware that final proof for primed ROS generation as the causal resistance mechanism underpinning IC1270-mediated ISR requires the use of inhibitor compounds able to abrogate the oxidative burst (e.g. DPI), such scavenger experiments could not be performed since detached leaves, needed for effective infiltration of chemicals in rice, somehow failed to develop ISR. Therefore, we can not rule out the possibility that the altered pathogen response of IC1270-induced plants may result in part from ROS-independent processes. Nonetheless, the involvement of boosted ROS generation in the establishment of IC1270-mediated ISR is apparent.

In accordance with previous studies [56,80], continuous generation of H_2_O_2 _in situ by infiltration of G/GO or 3-AT did not induce any detectable cell death per se, indicating that additional pathogen-induced signals are needed for expression of HR-like cell death. Indeed, current concepts suggest that death of host cells during the HR requires the poised production of nitric oxide (NO) and ROS, coupled to simultaneous suppression of the plant's antioxidant machinery [81-83]. In view of these data, it could be reasoned that IC1270-mediated priming for potentiated ROS generation might lower the threshold for activation of programmed cell death, thereby blocking the hemibiotroph *M. oryzae *in its initial biotrophic phase. In line with this concept, there is ample evidence demonstrating that early-produced H_2_O_2 _is a central signal leading to the elicitation of a wide range of blast-effective defenses, among which programmed cell death. Most tellingly, Kachroo and associates [84] reported a fungal glucose oxidase gene to sequentially induce H_2_O_2 _generation, rapid HR-like cell death and enhanced resistance against *M. oryzae *when ectopically expressed in young rice plants. On the other hand, it is not inconceivable that IC1270-mediated priming for H_2_O_2 _may tilt the ROS-controlled cellular life-or-death balance toward death, thereby facilitating subsequent tissue colonization by the necrotrophs *R. solani *and *C. miyabeanu*s. This notion is corroborated by recent observations demonstrating that IC1270 pretreatment has no marked impact on the early infection events in *C. miyabeanus*- or *R. solani*-challenged plants except for a substantial increase in the number of dying cells preceding the fungal growth front (De Vleesschauwer and Höfte, unpublished results). However, given the myriad defense-related plant responses modulated by ROS [52,53], other yet unidentified mechanisms also may play a role.

## Conclusion

In summary, our results favor a model whereby effective root colonization of rice by IC1270 locks plants into a pathogen-inducible program of boosted ROS generation and prompt execution of HR-like cell death at sites of attempted pathogen invasion, a mechanism which shows remarkable similarity with R protein-mediated ETI responses. Although highly effective against the hemibiotroph *M. oryzae*, halting the pathogen in its biotrophic phase, IC1270 pretreatment enhanced infection by the necrotrophs *R. solani *and *C. miyabeanus*, possibly by facilitating pathogen-triggered host cell death. Considering that defense responses effective against *M. oryzae *may not be effective against or even assist infection by *R. solani *and *C. miyabeanus*, our work underscores the importance of utilizing appropriate innate defense mechanisms when breeding for broad-spectrum rice disease resistance.

## Authors' contributions

DDV designed and carried out all experimental work and drafted the manuscript. LC participated in the design of the study and helped to edit the manuscript. MH conceived of the study, participated in its design and coordination and helped to draft the manuscript. All authors read and approved the final manuscript.

## Supplementary Material

Additional file 1 **Analysis of the H_2_O_2_-generating potential of glucose/glucose oxidase**. (**A**) In vitro generation of H_2_O_2 _by a mixture of glucose (G; 2 mM) and glucose oxidase (GO; 100 units ml^-1^) as revealed by DAB staining (1 mg ml^-1^). (**B**), Generation of apoplast-localized H_2_O_2 _in G/GO-amended sheath epidermal cells. Leaf sheaths were vacuum-infiltrated with DAB (1 mg ml^-1^) 1 h before being treated with a G/GO mixture (2 mM G/100 units GO ml^-1^). Picture was taken 3 h post G/GO application. Scale bar = 20 μm. Click here for file
