# Supplementary figures and images for: Differential effectiveness of Serratia plymuthica IC1270-induced systemic resistance against hemibiotrophic and necrotrophic leaf pathogens in rice
Source: BMC Plant Biol. 2009 Jan 22;9:9. doi: 10.1186/1471-2229-9-9 (PMC2650696; doi:10.1186/1471-2229-9-9)

## Slide 1
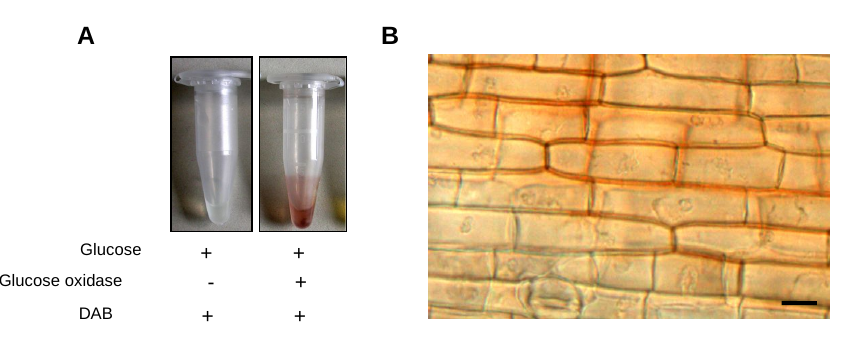

A
B
Glucose
 + +
 - +
Glucose oxidase
DAB
 + +

Supplement: Additional file 1 — Analysis of the H2O2-generating potential of glucose/glucose oxidase. (A) In vitro generation of H2O2 by a mixture of glucose (G; 2 mM) and glucose oxidase (GO; 100 units ml-1) as revealed by DAB staining (1 mg ml-1). (B), Generation of apoplast-localized H2O2 in G/GO-amended sheath epidermal cells. Leaf sheaths were vacuum-infiltrated with DAB (1 mg ml-1) 1 h before being treated with a G/GO mixture (2 mM G/100 units GO ml-1). Picture was taken 3 h post G/GO application. Scale bar = 20 μm. [file 1471-2229-9-9-S1.ppt]
